# Enhanced Lignocellulolytic Enzyme Activities on Hardwood and Softwood during Interspecific Interactions of White- and Brown-Rot Fungi

**DOI:** 10.3390/jof7040265

**Published:** 2021-03-31

**Authors:** Junko Sugano, Ndegwa Maina, Janne Wallenius, Kristiina Hildén

**Affiliations:** 1 Department of Microbiology, Faculty of Agriculture and Forestry, University of Helsinki, FI-00014 Helsinki, Finland; junko.sugano@helsinki.fi (J.S.); janne.wallenius@helsinki.fi (J.W.); 2 Department of Food and Nutrition, Faculty of Agriculture and Forestry, University of Helsinki, FI-00014 Helsinki, Finland; henry.maina@helsinki.fi

**Keywords:** wood decay, lignocellulose, carbohydrate active enzyme, laccase, white rot, brown rot, synergy

## Abstract

Wood decomposition is a sophisticated process where various biocatalysts act simultaneously and synergistically on biopolymers to efficiently break down plant cell walls. In nature, this process depends on the activities of the wood-inhabiting fungal communities that co-exist and interact during wood decay. Wood-decaying fungal species have traditionally been classified as white-rot and brown-rot fungi, which differ in their decay mechanism and enzyme repertoire. To mimic the species interaction during wood decomposition, we have cultivated the white-rot fungus, *Bjerkandera adusta*, and two brown-rot fungi, *Gloeophyllum sepiarium* and *Antrodia sinuosa*, in single and co-cultivations on softwood and hardwood. We compared their extracellular hydrolytic carbohydrate-active and oxidative lignin-degrading enzyme activities and production profiles. The interaction of white-rot and brown-rot species showed enhanced (hemi)cellulase activities on birch and spruce-supplemented cultivations. Based on the enzyme activity profiles, the combination of *B. adusta* and *G. sepiarium* facilitated birch wood degradation, whereas *B. adusta* and *A. sinuosa* is a promising combination for efficient degradation of spruce wood, showing synergy in β-glucosidase (BGL) and α-galactosidase (AGL) activity. Synergistic BGL and AGL activity was also detected on birch during the interaction of brown-rot species. Our findings indicate that fungal interaction on different woody substrates have an impact on both simultaneous and sequential biocatalytic activities.

## 1. Introduction

Plant biomass degradation is a complex process where various enzymes act simultaneously and synergistically on diverse biopolymers in order to efficiently breakdown plant cell walls. In natural habitats, this process strongly depends on the activities of microbial communities. In boreal and temperate forests, white-rot and brown-rot basidiomycete species play an important role in carbon cycling due to their ability to efficiently degrade or modify main lignocellulosic polymers present in the wood cell walls [1,2]. White-rot fungi have a unique ability to degrade all wood cell wall polymers, whereas brown-rot fungi hydrolyze cellulose and hemicelluloses, leaving modified, mainly demethoxylated, lignin behind.

The different wood degradation approaches rely on a diverse repertoire of oxidative and hydrolytic extracellular enzymes secreted by wood-rotting fungal species [3]. White-rot fungi are able to secrete a wide variety of carbohydrate active enzymes (CAZymes; www.cazy.org (accessed on 16 February 2021)) and lignin-modifying oxidoreductases (Table S1). White-rot fungi form the majority of wood-degrading basidiomycetes, and the most intensively studied species are commonly isolated from hardwoods [1], which have slightly higher cellulose and hemicellulose contents than softwoods [4]. White-rot species, *Bjerkandera adusta*, has the full repertoire of genes involved in cellulose and hemicellulose degradation. In addition, enzymes involved in lignin degradation, such as manganese peroxidase (MnP) and laccase, have been detected (Table S2). 

In contrast, brown-rot fungi produce only a limited set of lignocellulose depolymerizing enzymes [5]. Most brown rotters lack some cellulose-degrading enzymes, such as cellobiohydrolases (CBHs) [3,6], as well as lignin-modifying heme-peroxidases. Enzymatic hydrolysis of wood polysaccharides is enhanced by hydroxyl radicals via the non-enzymatic Fenton reaction [7,8]. Loss of wood strength is caused by the depolymerization of cellulose and hemicellulose, whereas lignin is only slightly modified [4]. *Antrodia sinuosa* and *Gloeophyllum sepiarium* are brown-rot species in the order Polyporales and Gloeophyllales, respectively. They are mainly found on decaying conifer trees, but they can also colonize hardwood [9,10,11,12,13]. In addition, they are both associated with degradation of wooden buildings [14]. *A. sinuosa* is an efficient cellulose degrader, and its genome includes gene models encoding cellulases and hemicellulases e.g., from glycoside hydrolase (GH) families 1, 2, 3, 5, 12 and 27, and auxiliary activities (AA) such as lytic polysaccharide monooxygenases and laccases (Table S2) [15]. *G. sepiarium* genome data is not available, but whole genome sequence and multi-omics data for a taxonomically related and well-studied model brown-rot species, *Gloeophyllum trabeum*, does exist [16,17,18]. *G. trabeum* has a typical repertoire of CAZyme-encoding genes detected in brown-rot species. On spruce wafers, *G. trabeum* has been reported to secrete endoglucanases, mannanases and xylanases. Both brown-rot species also have numerous gene models annotated to the CAZy family, AA3. The AA3 family includes enzymes from the glucose–methanol–choline oxidoreductase superfamily that catalyzes the formation of H_2_O_2_ for the Fenton reaction (Table S2).

Interaction between fungal species is fundamental in all stages of wood decay. Interspecific interactions have been studied in processes where the ecological roles of diverse species and their community dynamics have been in focus [19,20,21,22]. Although combative fungal interactions are common in nature, fungal co-occurrence and succession have also been observed on decaying wood [23,24]. The community structure, wood properties and different stages of wood decay have an impact on fungal enzyme activities, but only a few studies have targeted activity profiles of lignocellulosic substrates [25,26,27,28,29,30,31]. We have investigated the decay rate of wood species commonly found in Nordic boreal forests by cultivating white-rot and brown-rot fungi in different species combinations [32]. In this study, we investigated how the synergistically acting fungal species benefit from each other’s enzymatic abilities when they were cultivated on these different wood species. In addition, enzyme synergy between different combinations of white-rot and brown-rot fungal species was examined.

## 2. Materials and Methods

### 2.1. Fungal Cultures

Two Basidiomycota brown-rot fungal species, *Antrodia sinuosa* (CBS 142277) and *Gloeophyllum sepiarium* (CBS 142272), and a white-rot species, *Bjerkandera adusta* (CBS 142279), were pre-cultured on 2% (*w*/*v*) malt extract agar plates at 28 °C in the dark for 7 days. 100 mL of low-nitrogen asparagine–succinate medium (LN-AS, pH 4.5) [33] was supplemented with 1 g (dry weight) *Pinus sylvestris* (Scots pine), *Picea abies* (Norway spruce), *Betula pendula* (birch) sawdust or 0.1 g microcrystalline cellulose (Avicel^®^ PH-101; Sigma-Aldrich, St. Louis, MO, USA) as sole carbon sources. LN-AS liquid cultures were inoculated with 64 small (ca. 3 mm × 3 mm) agar pieces cut from pre-cultivated agar plates (2.5 cm × 2.5 cm area). The fungal isolates were cultivated in single cultivations and in two species co-cultivations with agitation (120 rpm) in the dark at 28 °C for 35 days. All cultivations were performed with three biological replicates.

### 2.2. Enzyme Assays

For enzyme activity measurements, 2 mL samples were taken twice a week from each cultivation flask. All the activities were calculated as µkat/L. Carbohydrate-active enzymes (CAZymes) and laccase (Lcc) activities were measured in 96-well microtiter plates using Tecan Spark version 1.2 (Tecan, Austria). β-1,4-Endoglucanase (EG), β-1,4-Endoxylanase (XLN) and β-1,4-Endomannanase (MAN) activities were detected using a dinitrosalicylic acid assay [34] in 50 mM Na-citrate buffer, pH 4.8. For EG, XLN and MAN activity, 1% (*w*/*v*) carboxymethyl cellulose (CMC; Sigma-Aldrich, USA), 1% (*w*/*v*) xylan from beechwood (Sigma, Neustadt, Germany) [35,36] and 0.5 % (*w*/*v*) locust bean gum (Sigma-Aldrich, USA) [34] were used as substrates, respectively. The absorbance was measured at 540 nm. 

Exo-acting enzyme activities, β-1,4-Glucosidase (BGL), β-1,4-Xylosidase (BXL), β-1,4-Mannosidase (MND) and α-1,4-Galactosidase (AGL) were determined using the 4-nitrophenyl (pNP) assay method at 405 nm with 0.1% of 4-nitrophenyl β-D-glucopyranoside, 4-nitrophenyl β-D-xylopyranoside, 4-nitrophenyl β-D-mannopyranoside and 4-nitrophenyl α-D-galactopyranoside, respectively, as substrates [37,38]. All pNP-linked substrates were purchased from Sigma-Aldrich (USA).

Cellobiohydrolase (CBHI) activity was measured using 2 mM 4-methylumbelliferyl-β-D-lactoside (MULac; Biokemis, Russia) in 50 mM sodium citrate buffer (pH 5.0) as a substrate. The reaction was stopped with 1 M Na-carbonate after incubation for 10 min at 45 °C, and the absorbance was measured at 370 nm. 

Lcc activity was determined at 476 nm by detecting the oxidation of 2,6-dimethoxyphenol (2,6-DMP; Aldrich, Darmstadt, Germany) in McIlvain buffer, pH 3.0 and 5.0 [39]. The activity was calculated as nkat/mL. Manganese peroxidase (MnP) activity was measured by following the formation of Mn^3+^-malonate complex at 270 nm in 0.05 M Na-malonate buffer, pH 4.5, with Shimadzu UV-1700 PharmaSpec (Kyoto, Japan) [40]. 

### 2.3. Statistical Analysis and Fungal Carbohydrate-Active Enzymes

Enzyme activities of the co-cultivations were compared with both individual cultivations by one-way analysis of variance (ANOVA) or the Kruskal–Wallis test to determine significance at 5% level (*p* < 0.05). The Tukey HSD or Steel–Dwass test was used for multiple comparisons using statistical software R Version 3.6.1 and RStudio Version 1.2.5001 [41]. Homogeneity of variances (*p* > 0.05) was tested using the Levene or Fligner–Killeen test. A *t*-test was conducted to compare sugar compositions between untreated wood and fungal-treated wood.

### 2.4. Substrate Composition Analysis

Sugar compositions derived from hemicellulose of natural wood sawdust, fungal cultured wood sawdust and fungal mycelia were analyzed by acid methanolysis methods [42,43]. Ten milligrams ± 0.4 mg of samples and sugar standards [arabinose (Ara), rhamnose (Rha), galactose (Gal), mannose (Man), galacturonic acid (GalA), glucuronic acid (GlcA), glucose (Glu) and xylose (Xyl)] were weighed in pear-shaped flasks with three replications. The samples were dried in a vacuum oven at 30 °C for 30 min. The samples were dissolved in 2 mL of an acid methanolysis reagent (2 M HCl in anhydrous methanol) and incubated at 100 °C for 5 h. After cooling to room temperature, 100 µL of pyridine was added to neutralize the samples. The solutions were filled up to 10 mL in the volumetric flasks. For the silylation, 100 µL of internal standard (0.1% of sorbitol in methanol) was added to 2 mL of the methanolysis samples. After evaporating the solutions under nitrogen gas at 50 °C, 100 µL of pyridine and 100 µL of silylation reagent (1:99 of trimethylsilyi chloride (TMSCI) and N,O-Bis(Trimethylsilyl) trifluoroacetamide (BSTFA)) were added. The samples were incubated at 60 °C for 30 min, followed by addition of 1 mL of heptane. 

Prior to gas chromatographic (GC) analysis, the samples were filtered (0.45 µm, Acrodisc), and 1 µL of each sample was injected into capillary column DB-1 (30 m/250 µm Internal Diameter, 0.25 µm filter thickness) in GC-MS 6890N (Agilent Technologies, California, USA) and measured at 250 °C, split ratio 20:1. The temperature program was started with 150 °C (3 min), a ramp of 2°C/min to 186°C followed by a ramp of 1 °C/min to 200 °C and 20 °C/min to 300 °C (1 min). The total run time was 40 min. A frame ionization detector (FID) was used at 280 °C. Helium was used as a carrier gas. All data were analyzed by a software, Agilent ChemStation (Agilent Technologies, Santa Clara, CA, USA). The dry weights of sugars were given as g/100 g wood.

## 3. Results

### 3.1. Hydrolytic Enzyme Activities

#### 3.1.1. Cellulolytic Activities

**Figure 1:**
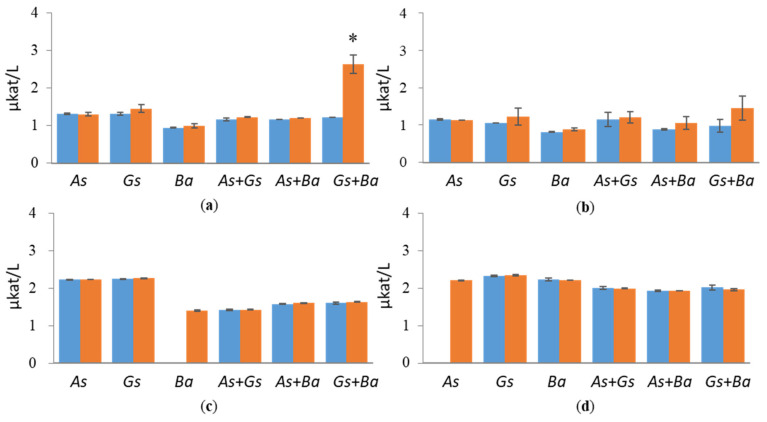
Endoglucanase (EG) activities at early (day 10, marked in blue) and late time points (day 24, marked in red) in single and co-cultivation on (**a**) birch, (**b**) spruce, (**c**) pine wood and (**d**) Avicel. *As* = *A. sinuosa*, *Gs* = *G. sepiarium*, *Ba* = *B. adusta*, *As*+*Gs* = *A. sinuosa* and *G. sepiarium*, *As*+*Ba* = *A. sinuosa* and *B. adusta*, *Gs+Ba* = *G. sepiarium* and *B. adusta*. Error bars refer to standard deviation (SD) (*n* = 3). * indicates statistical difference among single and co-cultivations (*p* < 0.05).

**Figure 2:**
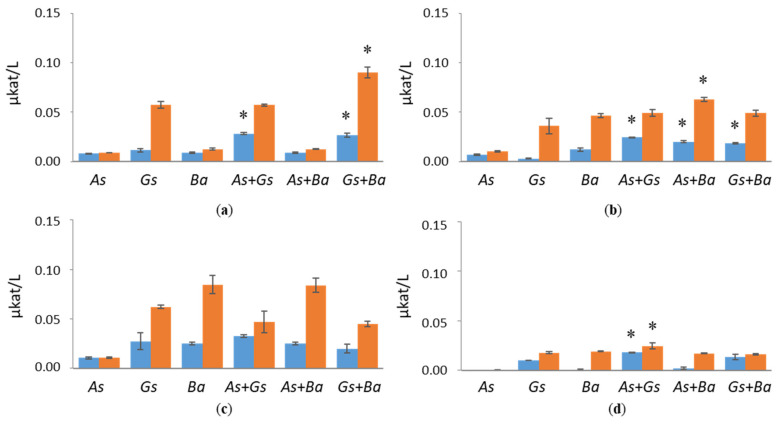
β-glucosidase (BGL) activities at early (day 10, marked in blue) and late time points (day 24, marked in red) in single and co-cultivation on (**a**) birch, (**b**) spruce, (**c**) pine wood and (**d**) Avicel. *As* = *A. sinuosa*, *Gs* = *G. sepiarium*, *Ba* = *B. adusta*, *As*+*Gs* = *A. sinuosa* and *G. sepiarium*, *As*+*Ba* = *A. sinuosa* and *B. adusta*, *Gs+Ba* = *G. sepiarium* and *B. adusta*. Error bars refer to standard deviation (SD) (*n* = 3). * indicates statistical difference among single and co-cultivations (*p* < 0.05).

All fungal strains were cultivated in a minimal medium supplemented with either wood sawdust or Avicel. Selected enzyme activities targeting cellulose and hemicellulose were determined from liquid cultivations twice a week. In cultivations with birch and pine, EG and BGL activities were slightly higher compared to spruce (Figure 1 and Figure 2 and Figure S1). EG activities stayed constant in all cultivation conditions. On day 24, co-cultivation of white-rot fungus, *B. adusta*, and brown-rot fungus, *G. sepiarium*, showed higher EG activity compared to single cultivations with birch (2.6 µkat/L) as carbon sources (Figure 1). On birch-supplemented conditions, towards the end of the cultivation period, EG activity was higher than with Avicel, which was prepared as a positive control. In the single cultivations, the highest activities (approx. 2.2 µkat/L) were detected in brown-rot species, *A. sinuosa* and *G. sepiarium*, with pine as a sole carbon source. 

Co-cultivated *B. adusta* and *G. sepiarium* indicated synergistic BGL activity both at early (0.03 µkat/L) and late time points (0.09 µkat/L) of cultivation on birch (Figure 2). Synergistic effects on BGL activity were also detected in *G. sepiarium* co-cultivations with *A. sinuosa* at day 10 on birch (0.03 µkat/L), which turned into additive effects towards the end of the cultivation (Figure S2). In *B. adusta*, BGL activity was the highest in softwood-containing cultivations, reaching 0.08 µkat/L on pine and 0.05 µkat/L on spruce (Figure 2). BGL activities were low in Avicel-containing cultivations throughout the cultivation period.

**Figure 3:**
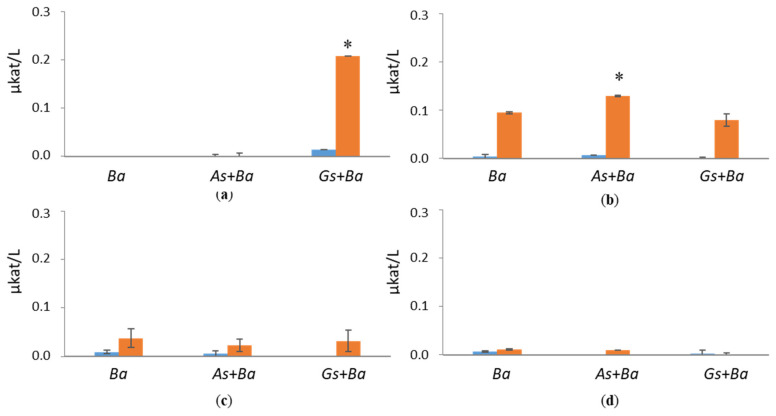
Cellobiohydrolase I (CBHI) activities at early (day 10, marked in blue) and late time points (day 24, marked in red) in single and co-cultivation on (**a**) birch, (**b**) spruce, (**c**) pine wood and (**d**) Avicel. *Ba* = *B. adusta*, *As*+*Ba* = *A. sinuosa* and *B. adusta*, *Gs+Ba* = *G. sepiarium* and *B. adusta*. Error bars refer to standard deviation (SD) (*n* = 3). * indicates statistical difference between *B. adusta* and co-cultivation with brown-rot species (*p* < 0.05).

CBHI activity was detected only in white-rot species, *B. adusta*, cultivations supplemented with softwood (Figure 3 and Figure S3). Surprisingly, co-cultivation of *B. adusta* together with brown-rot species, *A. sinuosa* or *G. sepiarium*, showed enhanced CBHI activity in the presence of birch (0.21 µkat/L) and spruce (0.13 µkat/L), respectively, at day 24 of the cultivation. However, in pine-containing growth conditions, co-cultivations reduced CBHI activity of *B. adusta*. CBHI activities were negligible in Avicel cultivation.

#### 3.1.2. Hemicellulolytic Activities

**Figure 4:**
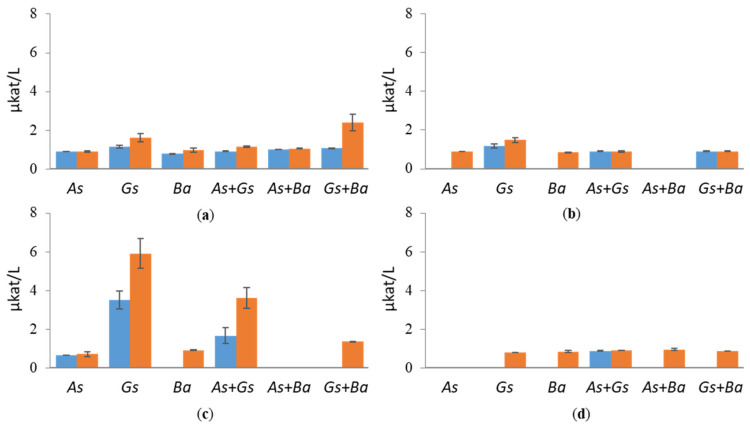
Endoxylanase (XLN) activities at early (day 10, marked in blue) and late time points (day 24, marked in red) in single and co-cultivation on (**a**) birch, (**b**) spruce, (**c**) pine wood and (**d**) Avicel. *As* = *A. sinuosa*, *Gs* = *G. sepiarium*, *Ba* = *B. adusta*, *As*+*Gs* = *A. sinuosa* and *G. sepiarium*, *As*+*Ba* = *A. sinuosa* and *B. adusta*, *Gs+Ba* = *G. sepiarium* and *B. adusta*. Error bars refer to standard deviation (SD) (*n* = 3). * indicates statistical difference among single and co-cultivations (*p* < 0.05).

**Figure 5:**
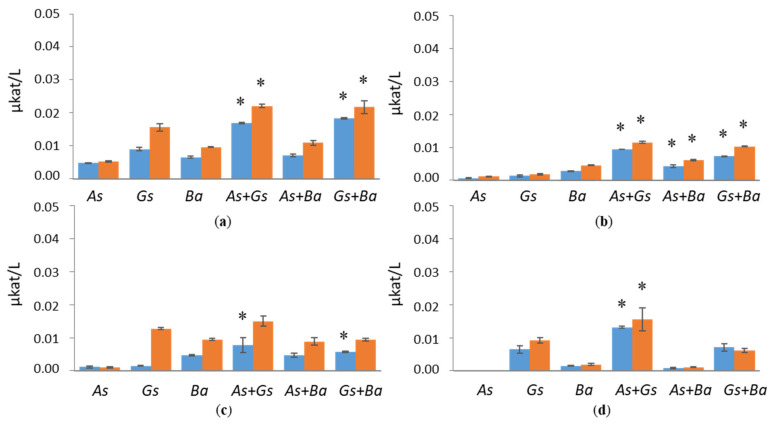
β-xylosidase (BXL) activities at early (day 10, marked in blue) and late time points (day 24, marked in red) in single and co-cultivation on (**a**) birch, (**b**) spruce, (**c**) pine wood and (**d**) Avicel. *As* = *A. sinuosa*, *Gs* = *G. sepiarium*, *Ba* = *B. adusta*, *As*+*Gs* = *A. sinuosa* and *G. sepiarium*, *As*+*Ba* = *A. sinuosa* and *B. adusta*, *Gs+Ba* = *G. sepiarium* and *B. adusta*. Error bars refer to standard deviation (SD) (*n* = 3). * indicates statistical difference among single and co-cultivations (*p* < 0.05).

Co-cultivation of *B. adusta* with *G. sepiarium* showed the highest XLN and BXL activities on birch (Figure 4 and Figure 5). XLN activity of the co-cultivation started to increase after three weeks, reaching 3.8 µkat/L on day 35 (Figure S4), and BXL activity showed a constant rise from day three (0.012 µkat/L) to the end of the cultivation (0.025 µkat/L) (Figure S5). The highest XLN activities in co-cultivations were detected in pine during the interaction of brown rotters, *G. sepiarium* and *A. sinuosa*, where activity started to increase after one week, reaching 4.5 µkat/L on day 28 (Figure S4). However, even higher XLN activities (6.2 µkat/L) were detected in a single cultivation of *G. sepiarium* (Figure 4). On birch-supplemented cultivations, XLN and BXL activities stayed constant throughout the cultivation period, whereas XLN activities in pine- and spruce-supplemented cultivations fluctuated. In Avicel, activities towards xylan were low in all cultivation conditions and species.

**Figure 6:**
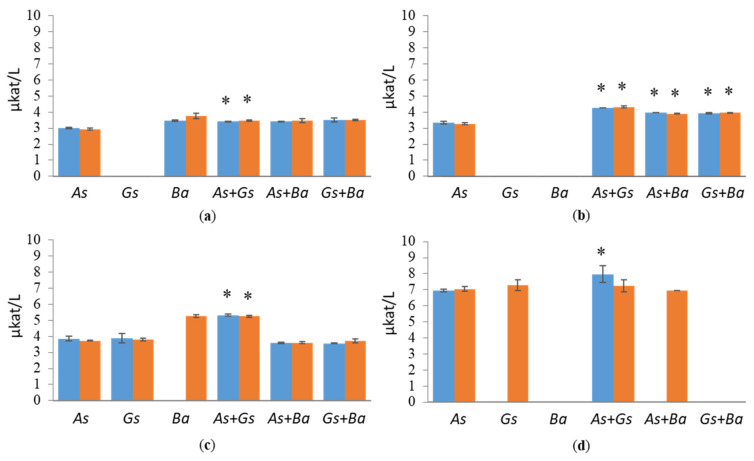
Endomannanase (MAN) activities at early (day 10, marked in blue) and late time points (day 24, marked in red) in single and co-cultivation on (**a**) birch, (**b**) spruce, (**c**) pine wood and (**d**) Avicel. *As* = *A. sinuosa*, *Gs* = *G. sepiarium*, *Ba* = *B. adusta*, *As*+*Gs* = *A. sinuosa* and *G. sepiarium*, *As*+*Ba* = *A. sinuosa* and *B. adusta*, *Gs+Ba* = *G. sepiarium* and *B. adusta*. Error bars refer to standard deviation (SD) (*n* = 3). * indicates statistical difference among single and co-cultivations (*p* < 0.05).

**Figure 7:**
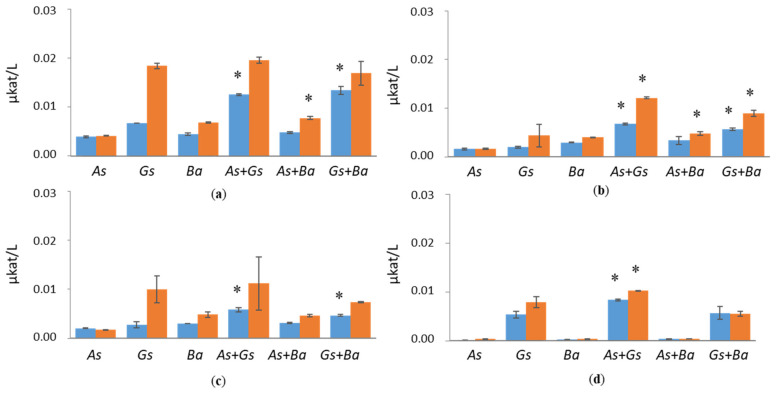
β-mannosidase (MND) activities at early (day 10, marked in blue) and late time points (day 24, marked in red) in single and co-cultivation on (**a**) birch, (**b**) spruce, (**c**) pine wood and (**d**) Avicel. *As* = *A. sinuosa*, *Gs* = *G. sepiarium*, *Ba* = *B. adusta*, *As*+*Gs* = *A. sinuosa* and *G. sepiarium*, *As*+*Ba* = *A. sinuosa* and *B. adusta*, *Gs+Ba* = *G. sepiarium* and *B. adusta*. Error bars refer to standard deviation (SD) (*n* = 3). * indicates statistical difference among single and co-cultivations (*p* < 0.05).

MAN and MND exhibited almost flat activities in all cultivation conditions (Figure 6 and Figure 7). MAN activities in pine-containing conditions showed fluctuation in all single and co-cultivations except in the *A. sinuosa* single cultivation, where MAN activity stayed approximately 3.8 µkat/L throughout the cultivation period (Figure S6). Co-cultivated *A. sinuosa* and *G. sepiarium* showed higher MAN and MND activities on most of the wood-supplemented cultivations compared to the single cultivations (Figure 6 and Figure S7). Surprisingly, for both brown-rot species, the highest MAN activities were detected on Avicel, whereas *B. adusta* showed no activity. 

**Figure 8:**
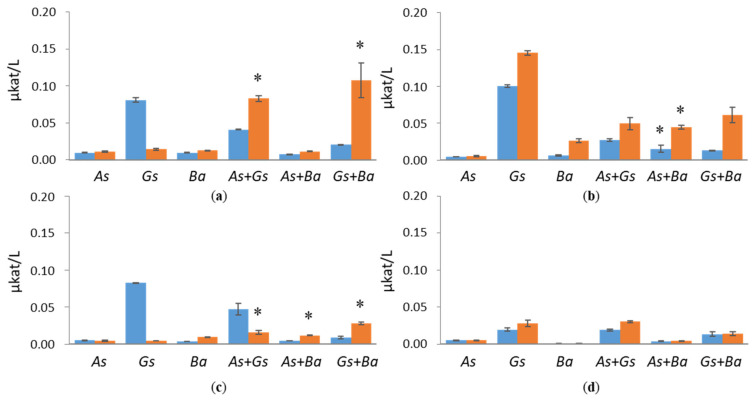
α-galactosidase (AGL) activities at early (day 10, marked in blue) and late time points (day 24, marked in red) in single and co-cultivation on (**a**) birch, (**b**) spruce, (**c**) pine wood and (**d**) Avicel. *As* = *A. sinuosa*, *Gs* = *G. sepiarium*, *Ba* = *B. adusta*, *As*+*Gs* = *A. sinuosa* and *G. sepiarium*, *As*+*Ba* = *A. sinuosa* and *B. adusta*, *Gs+Ba* = *G. sepiarium* and *B. adusta*. Error bars refer to standard deviation (SD) (*n* = 3). * indicates statistical difference among single and co-cultivations (*p* < 0.05).

The highest AGL activity was detected in the *G. sepiarium* single cultivation on spruce -containing medium, where the activity was constantly growing during the cultivation period, being 0.18 µkat/L on day 35 (Figure S8). On birch- and pine-supplemented cultivations, the activity peaked in the second week of cultivation, reaching 0.09 µkat/L. *G. sepiarium* co-cultivated either with *A. sinuosa* or *B. adusta* showed the highest activity on birch-containing medium (Figure 8). Both combinations showed synergistic activity at the end of the cultivation period (0.10 and 0.15 µkat/L; Figure S8). Synergy was also detected in co-cultivated *A. sinuosa* and *B. adusta* on spruce (0.05 µkat/L), whereas the corresponding single cultivations showed activities below 0.008 and 0.03 µkat/L, respectively. Both MND and AGL activities were low on Avicel (Figure 7 and Figure 8). *G. sepiarium* showed low AGL activity in a single cultivation and together with *A. sinuosa*.

### 3.2. Activities of Lignin-Degrading Enzymes

**Figure 9:**
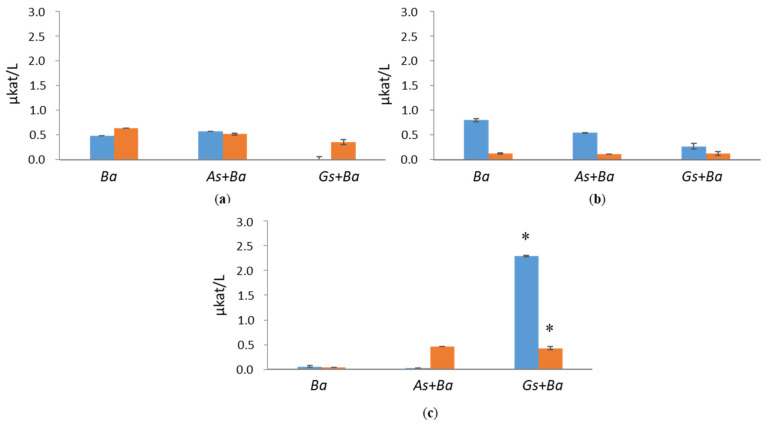
Manganese peroxidase (MnP) activities at early (day 10, marked in blue) and late time points (day 24, marked in red) in single and co-cultivation on (**a**) birch, (**b**) spruce and (**c**) pine wood. *Ba* = *B. adusta*, *As*+*Ba* = *A. sinuosa* and *B. adusta*, *Gs+Ba* = *G. sepiarium* and *B. adusta*. Error bars refer to standard deviation (SD) (*n* = 3). * indicates statistical difference among single and co-cultivations (*p* < 0.05).

**Figure 10:**
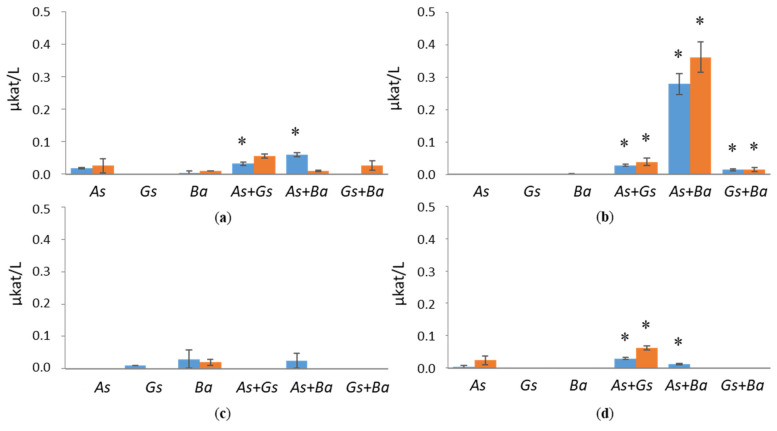
Laccase (Lcc) activities at early (day 10, marked in blue) and late time points (day 24, marked in red) in single and co-cultivation on (**a**) birch, (**b**) spruce, (**c**) pine wood and (**d**) Avicel. *As* = *A. sinuosa*, *Gs* = *G. sepiarium*, *Ba* = *B. adusta*, *As*+*Gs* = *A. sinuosa* and *G. sepiarium*, *As*+*Ba* = *A. sinuosa* and *B. adusta*, *Gs+Ba* = *G. sepiarium* and *B. adusta*. Error bars refer to standard deviation (SD) (*n* = 3). * indicates statistical difference among single and co-cultivations (*p* < 0.05).

In general, activities related to lignin degradation (MnP and Lcc) were low (Figure 9 and Figure 10). In *B. adusta*, the highest MnP activity was detected on the spruce-containing cultivation at day 7 (1.2 µkat/L; Figure S9). However, when *B. adusta* was co-cultivated with *G. sepiarium*, MnP activity on pine showed 2.3 µkat/L on day 10 (Figure 9). Lcc activities in all single cultivations were negligible. Only co-cultivated *B. adusta* and *A. sinuosa* showed low but clear activity on spruce with a synergistic potential (0.4 µkat/L; Figure 10). 

### 3.3. Wood Composition after Fungal Treatment

The chemical composition of birch, spruce and pine was analyzed before and after fungal treatment (Table 1). In untreated softwoods, the most abundant hemicellulose-derived monosaccharide was mannose (10.8 % in pine and 10.3 % in spruce), whereas xylose dominated in birch (21.4 %). The amount of glucomannan-derived monosaccharides i.e., mannose (Man) and glucose (Glu), in pine were significantly reduced after fungal treatment, as well as pectin-derived GalA. Only on the *A. sinuosa* single cultivation, the reduction was not statistically significant. In contrast, in spruce-containing cultivations, the amount of mannan-derived monosaccharides were not affected by fungal treatment. On birch, the xylan-derived monosaccharide, xylose (Xyl), was significantly decreased in co-cultivated *A. sinuosa* and *B adusta*, as well as in the single cultivation of *B. adusta*. Both brown-rot species in single and co-cultivations showed significant losses of Glu derived from hemicelluloses in birch. 

## 4. Discussion

In natural habitats, plant biomass degradation strongly depends on the activities of fungal communities. The complete and efficient decay requires the combined and diverse activities of multiple fungal species that act simultaneously and synergistically. We have studied lignocellulose-degrading enzyme activities of fungal species, which have been previously shown to be able to grow together on carboxymethyl cellulose (CMC)-supplemented agar plates [15]. This decay community included two brown-rot fungi (*G. sepiarium* and *A. sinuosa*) and one white-rot fungus (*B. adusta*), which we co-cultivated on wood-supplemented liquid cultivations. 

### 4.1. Co-Cultivation of White-Rot Fungus, B. adusta, and Brown-Rot Fungus, G. sepiarium

White-rot fungal species, such as *B. adusta*, are able to degrade all structural components of the plant cell wall, including lignin [44,45]. Although white-rot fungi are predominantly found in hardwood, *B. adusta* is able to colonize and degrade softwood as well [46,47,48,49]. In this study, *B adusta* and *G. sepiarium* were able to co-exist on softwood; however, most of the additive activities of hydrolytic enzymes were detected in hardwood (birch)-containing co-cultivations. Additionally, synergistic activities of BGL and AGL in co-cultivations were detected on birch-supplemented conditions. In contrast, in the pine- or spruce-containing co-cultivations, BGL activity was not enhanced by fungal interactions. Previously, variation in BGL activity has been detected in conifer-containing interspecific cultivations of brown-rot species, *Fomitopsis pinicola*, growing together with different white-rot species [29].

In single cultivations, both *B. adusta* and *G. sepiarium* showed only moderate EG activities on birch and spruce, whereas in pine, the activities were somewhat higher. Previously, it has been shown that *B. adusta* produced (hemi)cellulolytic enzymes e.g., GH1, GH3 and GH31, as well as lignin-modifying class II peroxidases during growth on beech wood (*Fagus sylvatica*) [50]. However, the enzyme activities have not been measured in this proteome study. To be able to follow a wide repertoire of enzyme activities during the 35-day cultivation, we have grown the fungi in wood-supplemented submerged cultivations, which cannot be directly compared with the proteome from solid wood. Similarly to *B. adusta* and *G. sepiarium*, single cultivations of white-rot fungus, *Phlebia radiata*, and brown rotter, *F. pinicola*, have shown moderate EG activity when growing on birch [51]. For *B. adusta* and *G. sepiarium*, the EG activity curve stayed flat for 35 days, whereas *P. radiata* and brown rotter, *F. pinicola*, have shown fluctuating EG activity during the cultivation.

CBHI activities of *B. adusta* were low overall, but on birch-supplemented co-cultivation, CBHI activity was significantly enhanced on day 24 compared to other wood substrates or single cultivations. In line with our study, spruce-containing cultivations of white-rot species, *Phlebia centrifuga* and *Dichomitus squalens*, have shown only low CBH activities [52,53].

In nature, *G. sepiarium* colonizes conifer trees [12], but here we show that *G. sepiarium* grows on birch as well. On birch-supplemented cultivations, xylan degradation-related enzyme activities were enhanced and xylose consumption was detected on *B. adusta* and *G. sepiarium* co-cultivations. High MAN and MND activities were detected on all wood supplemented cultivations in both single and co-cultivations of *B. adusta* and *G. sepiarium*. This is in line with the reduction of mannose and glucose residues in pine and spruce cultivations, which indicates consumption of these monosaccharides by fungi. Interestingly, MAN activities on pine fluctuated during the cultivation, whereas on spruce and birch cultivations, activities were constant throughout the cultivation period. Similarly, MND activities stayed constant in softwood cultivations, and on birch, there was a slight increase in activity during the cultivation period. In the related species, *G. trabeum*, high levels of secreted GH10 (xylanases) and GH5 (mannanases) have been detected on spruce wafers [54].

In brown-rot species, non-enzymatic (hemi)cellulose degradation is efficient during the early stages of wood decay [55]. However, high xylanase activity was detected for *G. sepiarium* in wood cultivations. In line with our results, XLN activity has been reported to be superior compared to cellulolytic enzyme activities in *G. trabeum* cultures on pine (*Pinus taeda*) wood chips [56].

Pectin, a minor component in wood, is present at high levels in bordered pits, which are often the entry points for fungal hyphae to the wood cell walls. We detected pectin-derived monosaccharides i.e., galacturonic acid, rhamnose, arabinose and galactose, but there was no clear pattern in their consumption by fungi.

In pine, MnP activity of *B. adusta* was enhanced by the presence of *G. sepiarium*, which does not have MnP-encoding genes in its genome (Table S2). Previously, enhanced MnP activity has been detected on co-cultivation of *B. adusta* and white-rot fungus *D. squalens* [57]. This indicates that MnP production of *B. adusta* is influenced by the co-existence of other fungal species.

### 4.2. Co-Cultivation of White-Rot Fungus, B. adusta, and Brown-Rot Fungus, A. sinuosa

Co-cultivations of *B. adusta* with brown-rot species, *A. sinuosa*, showed a different pattern of secreted enzyme activities compared to those detected in co-cultivations with *G. sepiarium.* The sequenced genome of *A. sinuosa* indicates that it has ability to breakdown both cellulose and hemicellulose (Table S2). In general, enzyme profiles of *B. adusta* and *A. sinuosa* co-cultivations showed no additive effect on softwood-containing cultivations. Only BGL and AGL showed synergistic activity on spruce, and the enzyme activity profile resembled the single cultivation of *B. adusta*. In single cultivations, *A. sinuosa* showed constant EG activity on all substrates, which has been also previously detected on CMC-supplemented liquid cultivations [15].

The additive effects of enzyme activities were observed for xylan- and mannan-acting enzymes on birch- and spruce-supplemented co-cultivations, respectively, indicating a positive interaction of these species in hemicellulose degradation. This is in line with the reduction of xylose residues in birch, and mannose and glucose residues in spruce after fungal treatment.

In single cultivations, XLN activity of *B. adusta* was produced towards the end of the cultivation in softwood. Similarly, xylanolytic activity of *B. adusta* has been detected in softwood- and hardwood-supplemented agar plates after two-week cultivations [49]. Xylanolytic activities of *Antrodia* species have been previously reported from *Antrodia macra* and *Antrodia pulvinascens*, where low XLN and high BXL activities have been measured in defined liquid cultivations [58].

### 4.3. Co-Cultivation of Brown-Rot Fungi, G. sepiarium and A. sinuosa

The interaction of taxonomically different brown-rot fungal species, *A. sinuosa* and *G. sepiarium*, indicated that the enzyme-production profile was affected by the co-existence on wood. In nature, this combination is rare, but a positive effect on degradation of spruce has been detected in the three species co-cultivation with white-rot species, *Phlebiopsis gigantea* [32]. We cultivated these brown-rot species on spruce and pine, as well as on birch, which is not a natural substrate for either of these brown-rot species. However, species in the Antrodia–Fomitopsis clade have been suggested to be generalists in degradation of both angiosperms and gymnosperm species in nature [59].

At early stages of co-cultivation, synergy in BGL production was detected on birch and spruce. BXL activity was enhanced in all substrates in co-cultivations, whereas XLN activity of *G. sepiarium* was inhibited by the presence of *A. sinuosa*. The reduced amount of xylose was detected only in birch-containing cultivations. In line with our findings, the interaction of *G. trabeum*, which is a related species to *G. sepiarium*, and *Rhodonia placenta*, which belongs to the same clade as *A. sinuosa*, have shown lower XLN activity in the interaction zone compared with single cultivations or co-cultivation outside the interacting hyphae, when grown on aspen [18]. However, no synergy or elevated production of BGL was detected in the interaction zone of *G. trabeum* and *R. placenta*.

In softwood, mannanolytic activities were higher in co-cultivations compared to single ones, which could also be observed in the reduced amount of mannose and glucose in fungal-treated spruce and pine. However, AGL activities were inhibited on softwood, whereas on pine- and birch-supplemented cultivations, the interaction of *A. sinuosa* and *G. sepiarium* caused synergistic activity at the later stages of the cultivation period.

Laccase activity was slightly enhanced on spruce and birch, which may indicate elevated stress in co-cultivations. Induced laccase activity in interspecific interactions has been reported to be related to the enhanced formations of melanin, which is suggested to protect the fungus against hydrolytic enzymes in the interaction zone [60]. In addition, laccase activity plays a defensive role in stressful conditions by degrading toxic metabolites during antagonistic fungal interactions [25,61].

In natural wood-inhabiting fungal communities, the interspecific interactions are dynamic processes [20]. The community development is dependent on both wood species and abiotic factors; therefore, the same combinations of fungal species may result in different interaction outcomes [62]. In addition, priority effects in the early stages of colonization, the ability to produce secondary metabolites in competitive interactions and the spatial orientation of fungal species have an impact on the fungal interaction in nature [60]. The different lignocellulose-modifying enzyme activities produced by positively interacting fungal species increase heterogeneity in the structure of degrading wood, which benefits utilization of nutrient resources by fungi.

## 5. Conclusions

To study enzymatic wood decay, we co-cultivated white-rot and brown-rot species in pairwise combinations in the presence of hardwood or softwood sawdust as a carbon source. The interaction of white-rot and brown-rot species showed enhanced (hemi)cellulase activities on birch- and spruce-supplemented cultivations. Based on the enzyme activity profiles and xylan degradation, the combination of *B. adusta* and *G. sepiarium* facilitated birch wood degradation, whereas *B. adusta* and *A. sinuosa* is a promising combination for efficient degradation of spruce wood. Our findings indicate that interspecific interactions of fungal species have a positive effect on the enzymatic degradation of wood.

## Figures and Tables

**Table 1:** Hemicellulose- and pectin-derived monosaccharide composition (g/100 g wood (%)) after fungal cultivations, measured by acid methanolysis. *As*+*Gs* = *A. sinuosa* and *G. sepiarium*, *As*+*Ba* = *A. sinuosa* and *B. adusta*, *Gs*+*Ba* = *G. sepiarium* and *B. adusta*.

		Man	Glu	Xyl	Gal	GalA	Ara	Rha	GlcA
Pine wood		10.76	9.98	5.72	2.31	1.37	1.36	0.44	n.d
Pine cultured with	*A. sinuosa*	9.13	7.38	7.51	2.53	1.22	1.62	0.48	n.d
	*G. sepiarium*	8.47 *	7.30 *	6.57 *	2.15	1.13 *	1.45	0.45	n.d
	*B. adusta*	6.22 *	6.07 *	5.77	2.22	1.11 *	1.22	0.45	n.d
	*As* + *Gs*	8.03 *	6.75 *	6.20	2.37	1.07 *	1.40	0.46	n.d
	*As* + *Ba*	6.53 *	6.41 *	5.99	3.19 *	1.19 *	1.23	0.46	n.d
	*Gs* + *Ba*	6.73 *	5.68 *	6.07	2.75 *	1.12 *	1.34	0.46	n.d
Spruce wood		10.30	3.84	4.42	2.13	1.46	0.79	0.42	n.d
Spruce cultured with	*A. sinuosa*	10.30	3.14	5.84 *	1.70	1.72	0.96	0.66 *	n.d
	*G. sepiarium*	10.61	3.34	5.94	2.03	1.64	0.99	0.65 *	n.d
	*B. adusta*	9.45	2.95	5.36	1.93	1.57	0.89	0.67 *	n.d
	*As* + *Gs*	9.59	3.05	5.13	1.89	1.55	0.85	0.63 *	n.d
	*As* + *Ba*	9.69	3.03	5.61 *	1.78	1.58	0.99	0.63 *	n.d
	*Gs* + *Ba*	9.42	3.06	5.49	1.95	1.62	0.94	0.64 *	n.d
Birch wood		0.98	7.56	21.44	1.17	2.16	0.53	0.68	0.35
Birch cultured with	*A. sinuosa*	1.23 *	5.28 *	24.15 *	1.74	2.11	0.69 *	0.79 *	n.d
	*G. sepiarium*	1.17 *	5.16 *	22.18	1.46 *	1.98	0.63 *	0.74 *	n.d
	*B. adusta*	1.74 *	10.47 *	13.19 *	2.33 *	1.85	n.d	0.60	n.d
	*As* + *Gs*	1.11	4.73 *	18.34	1.93 *	1.81	0.60	0.66	n.d
	*As* + *Ba*	1.85 *	10.18	11.69 *	3.86 *	1.67 *	0.58	0.56 *	n.d
	*Gs* + *Ba*	1.34	4.83	18.04	2.91	2.08	0.63	0.70	n.d

* indicates statistical difference between untreated and fungal treated wood (*p* < 0.05). n.d = not detected.

## Data Availability

Not applicable.

## Supplementary Materials

The following are available online at https://www.mdpi.com/article/10.3390/jof7040265/s1, Table S1: (Hemi) cellulose and lignin degrading enzymes; Table S2: Number of putative CAZyme encoding gene models in the genomes of *B. adusta*, *A. sinuosa* and *G. trabeum*; Figure S1: Endoglucanase (EG) activities on woody substrates and Avicel in single cultivations, a) *A. sinuosa*, b) *G. sepiarium*, and c) *B. adusta*, and in co-cultivations, d) *A. sinuosa* and *G. sepiarium*, e) *A. sinuosa* and *B. adusta*, and f) *G. sepiarium* and *B. adusta*. Error bars refer to standard deviation (SD) (*n* = 3); Figure S2: β-glucosidase (BGL) activities on woody substrates and Avicel in single cultivations, a) *A. sinuosa*, b) *G. sepiarium*, and c) *B. adusta*, and in co-cultivations, d) *A. sinuosa* and *G. sepiarium*, e) *A. sinuosa* and *B. adusta*, and f) *G. sepiarium* and *B. adusta*. Error bars refer to standard deviation (SD) (*n* = 3); Figure S3: Cellobiohydrolase I (CBHI) activities on woody substrates and Avicel in single cultivation of a) *B. adusta*, and in co-cultivations, b) *A. sinuosa* and *B. adusta*, and c) *G. sepiarium* and *B. adusta*. Error bars refer to standard deviation (SD) (*n* = 3); Figure S4: Endoxylanase (XLN) activities on woody substrates and Avicel in single cultivations, a) *A. sinuosa*, b) *G. sepiarium*, and c) *B. adusta*, and in co-cultivations, d) *A. sinuosa* and *G. sepiarium*, e) *A. sinuosa* and *B. adusta*, and f) *G. sepiarium* and *B. adusta*. Error bars refer to standard deviation (SD) (*n* = 3); Figure S5: β-xylosidase (BXL) activities on woody substrates and Avicel in single cultivations, a) *A. sinuosa*, b) *G. sepiarium*, and c) *B. adusta*, and in co-cultivations, d) *A. sinuosa* and *G. sepiarium*, e) *A. sinuosa* and *B. adusta*, and f) *G. sepiarium* and *B. adusta*. Error bars refer to standard deviation (SD) (*n* = 3); Figure S6: β-xylosidase (BXL) activities on woody substrates and Avicel in single cultivations, a) *A. sinuosa*, b) *G. sepiarium*, and c) *B. adusta*, and in co-cultivations, d) *A. sinuosa* and *G. sepiarium*, e) *A. sinuosa* and *B. adusta*, and f) *G. sepiarium* and *B. adusta*. Error bars refer to standard deviation (SD) (*n* = 3); Figure S7: β-mannosidase (MND) activities on Avicel and woody substrates in single cultivations, a) *A. sinuosa*, b) *G. sepiarium*, and c) *B. adusta*, and in co-cultivations, d) *A. sinuosa* and *G. sepiarium*, e) *A. sinuosa* and *B. adusta*, and f) *G. sepiarium* and *B. adusta*. Error bars refer to standard deviation (SD) (*n* = 3); Figure S8: α-galactosidase (AGL) activities on woody substrates and Avicel in single cultivations, a) *A. sinuosa*, b) *G. sepiarium*, and c) *B. adusta*, and in co-cultivations, d) *A. sinuosa* and *G. sepiarium*, e) *A. sinuosa* and *B. adusta*, and f) *G. sepiarium* and *B. adusta*. Error bars refer to standard deviation (SD) (*n* = 3); Figure S9: Manganese peroxidase (MnP) activities on woody substrates and Avicel in single cultivations of a) *B. adusta*, and in co-cultivations, b) *A. sinuosa and B. adusta*, and c) *G. sepiarium and B. adusta*. Error bars refer to standard deviation (SD) (*n* = 3)
