# Supplementary material for: Enhanced Lignocellulolytic Enzyme Activities on Hardwood and Softwood during Interspecific Interactions of White- and Brown-Rot Fungi
Source: J Fungi (Basel). 2021 Mar 31;7(4):265. doi: 10.3390/jof7040265 (PMC8065597; doi:10.3390/jof7040265)
Supplement: Supplementary file 1 [file jof-07-00265-s001.pdf]

Journal of Fungi

Supplementary material

Enhanced Lignocellulolytic Enzyme Activities on HARDWOod and Softwood during Interspecific Interactions of White and Brown Rot Fungi

Sugano Junko [1] Ndegwa Maina[2], Janne Wallenius[1] and Kristiina Hildén[1,*]

[1]Department of Microbiology, Faculty of Agriculture and Forestry, University of Helsinki, Finland junko.sugano@helsinki.fi; janne.wallenius@helsinki.fi; kristiina.s.hilden@helsinki.fi
[2]Department of Food and Nutrition, Faculty of Agriculture and Forestry, University of Helsinki, Finland henry.maina@helsinki.fi

* Correspondence: kristiina.s.hilden@helsinki.fi

Table S1. (Hemi)cellulose and lignin degrading enzymes

CAZy family(ies)	Enzyme abbreviation(s)	Activity	EC number	Substrate
GH1, 3	BGL	β-1,4-D-Glucosidase	3.2.1.21	Cellulose, Xyloglucan, Galactoglucomannan
GH2	MND	β-1,4-D-Mannosidase	3.2.1.25	Galactomannan
GH3, 39, 43	BXL	β-1,4-D-Xylosidase	3.2.1.37	Xylan
GH5, 12, 45, 131	EG	β-1,4-D-Endoglucanase	3.2.1.4	Cellulose
GH5_7, 26, 134	MAN	β-1,4-D-Endomannanase	3.2.1.78	Galactomannan
GH6, 7	CBHI	Cellobiohydrolase I	3.2.1.176	Cellulose
GH10, 11	XLN	β-1,4-D-Endoxylanase	3.2.1.8	Xylan
GH27, 36	AGL	α-1,4-D-Galactosidase	3.2.1.22	Xylan, Xyloglucan, Galactomannan
AA1	Lcc	Laccase		lignin
AA2	MnP, Lip, VP	Class II heme-peroxidases		lignin

Table S2. Number of putative CAZyme encoding gene models in the genomes of *B. adusta, A. sinuosa* and *G. trabeum.*

CAZy family			
Cellulose active	*B. adusta*	*A. sinuosa*	*G. trabeum*
GH1	2	1	5
GH3	9	3	11
GH5_5	4	2	2
GH5_22	2	2	2
GH6	1	0	0
GH7	5	0	0
GH9	1	0	1
GH12	2	13	2
GH45	1	0	1
GH131	3	0	1
Hemicellulose active			
GH2	3	3	4
GH5_7	2	2	2
GH10	4	2	3
GH11	0	0	0
GH27	3	2	3
GH29	0	0	1
GH31	4	5	5
GH35	4	1	2
GH43	6	1	6
GH51	2	1	4
GH74	2	0	1
GH95	1	1	1
GH115	2	1	2
Auxiliary activities			
AA1	2	7	5
AA2	21	1	0
AA3	39	21	24
AA9	28	2	4
AA14	3	2	2

http://genome.jgi.doe.gov/Antsi1/Antsi1.home.html; http://genome.jgi-psf.org/Glotr1_1/Glotr1_1.home.html; http://genome.jgi.doe.gov/Bjead1_1/Bjead1_1.home.html

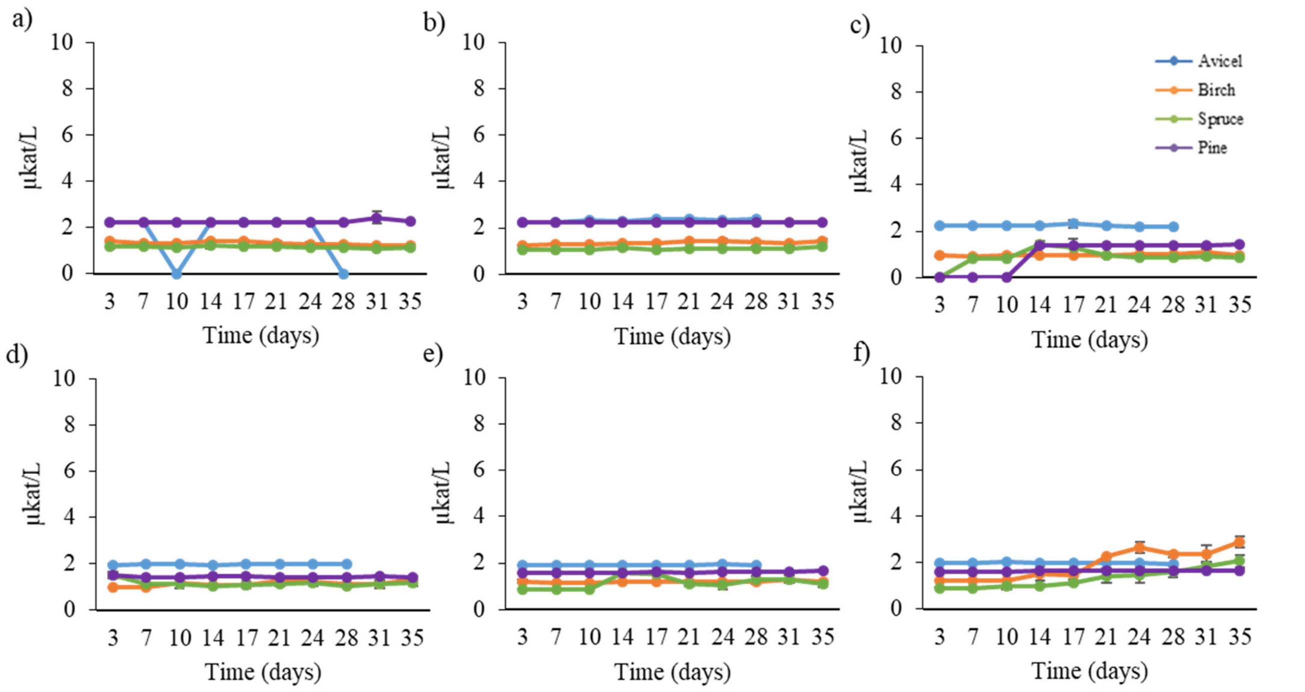

Figure S1. Endoglucanase (EG) activities on woody substrates and Avicel in single cultivations, a) *A. sinuosa*, b) *G. sepiarium*, and c) *B. adusta*, and in co-cultivations, d) *A. sinuosa* and *G. sepiarium*, e) *A. sinuosa* and *B. adusta*, and f) *G. sepiarium* and *B. adusta*. Error bars refer to standard deviation (SD) (n = 3

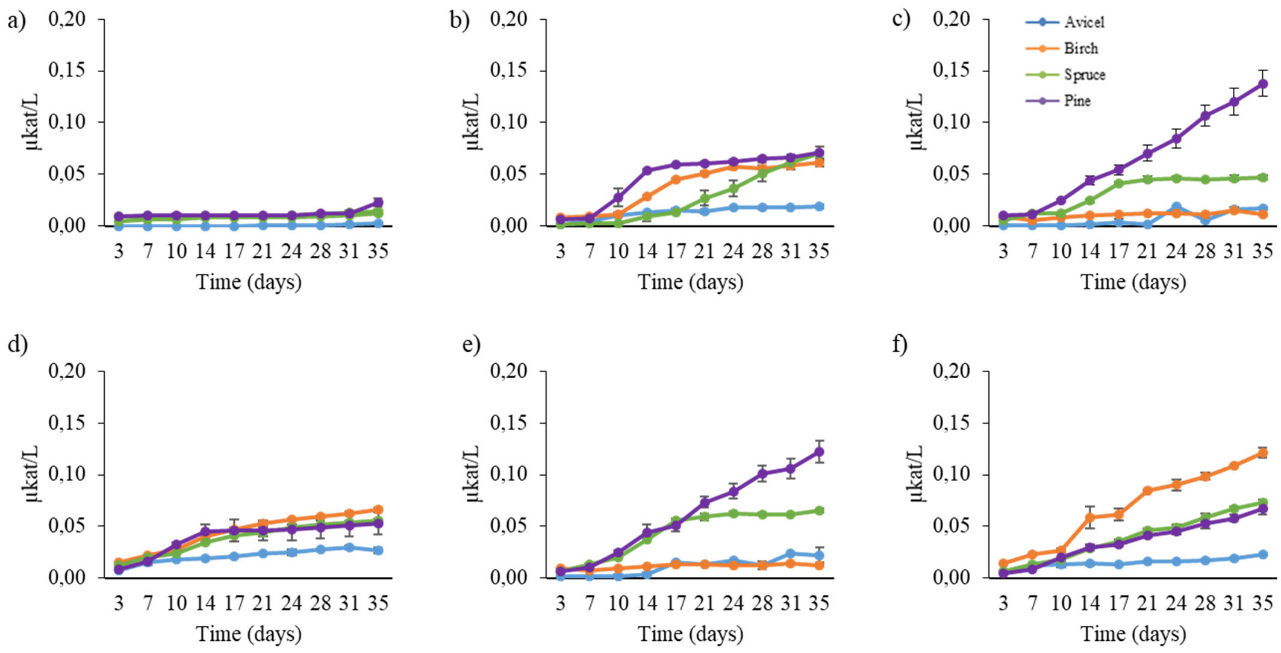

Figure S2. β-glucosidase (BGL) activities on woody substrates and Avicel in single cultivations, a) *A. sinuosa*, b) *G. sepiarium*, and c) *B. adusta*, and in co-cultivations, d) *A. sinuosa* and *G. sepiarium*, e) *A. sinuosa* and *B. adusta*, and f) *G. sepiarium* and *B. adusta*. Error bars refer to standard deviation (SD) (n = 3).

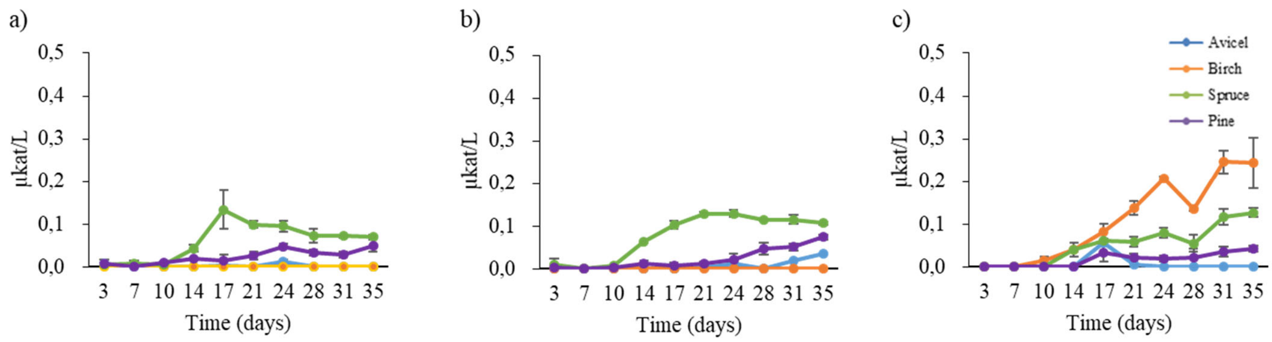

Figure S3. Cellobiohydrolase I (CBHI) activities on woody substrates and Avicel in single cultivation of a) *B. adusta*, and in co-cultivations, b) *A. sinuosa* and *B. adusta*, and c) *G. sepiarium* and *B. adusta*. Error bars refer to standard deviation (SD) (n = 3).

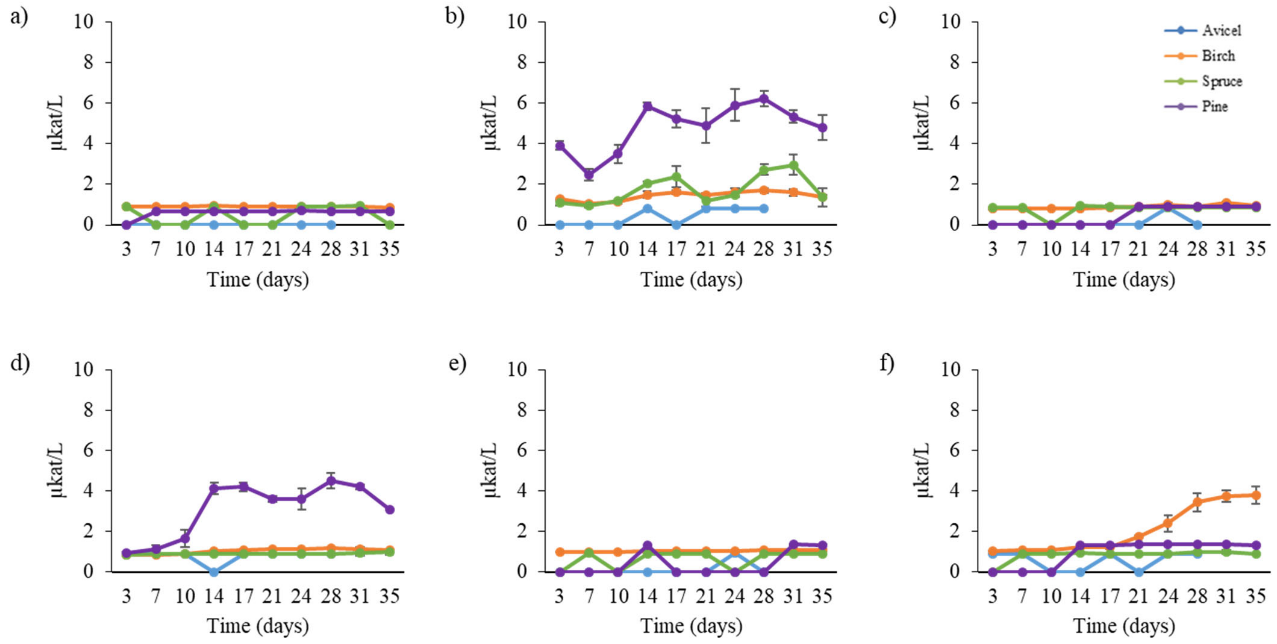

Figure S4. Endoxylanase (XLN) activities on woody substrates and Avicel in single cultivations, a) *A. sinuosa*, b) *G. sepiarium*, and c) *B. adusta*, and in co-cultivations, d) *A. sinuosa* and *G. sepiarium*, e) *A. sinuosa* and *B. adusta*, and f) *G. sepiarium* and *B. adusta*. Error bars refer to standard deviation (SD) (n = 3).

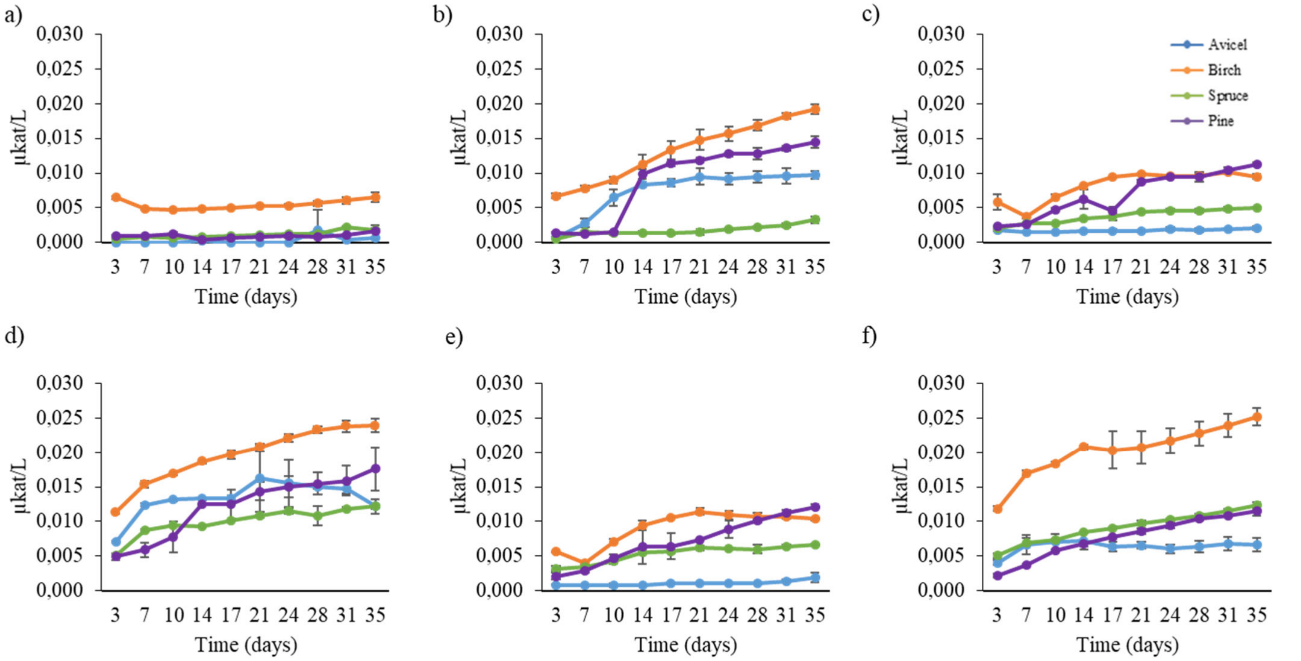

Figure S5. β-xylosidase (BXL) activities on woody substrates and Avicel in single cultivations, a) *A. sinuosa*, b) *G. sepiarium*, and c) *B. adusta*, and in co-cultivations, d) *A. sinuosa* and *G. sepiarium*, e) *A. sinuosa* and *B. adusta*, and f) *G. sepiarium* and *B. adusta*. Error bars refer to standard deviation (SD) (n = 3).

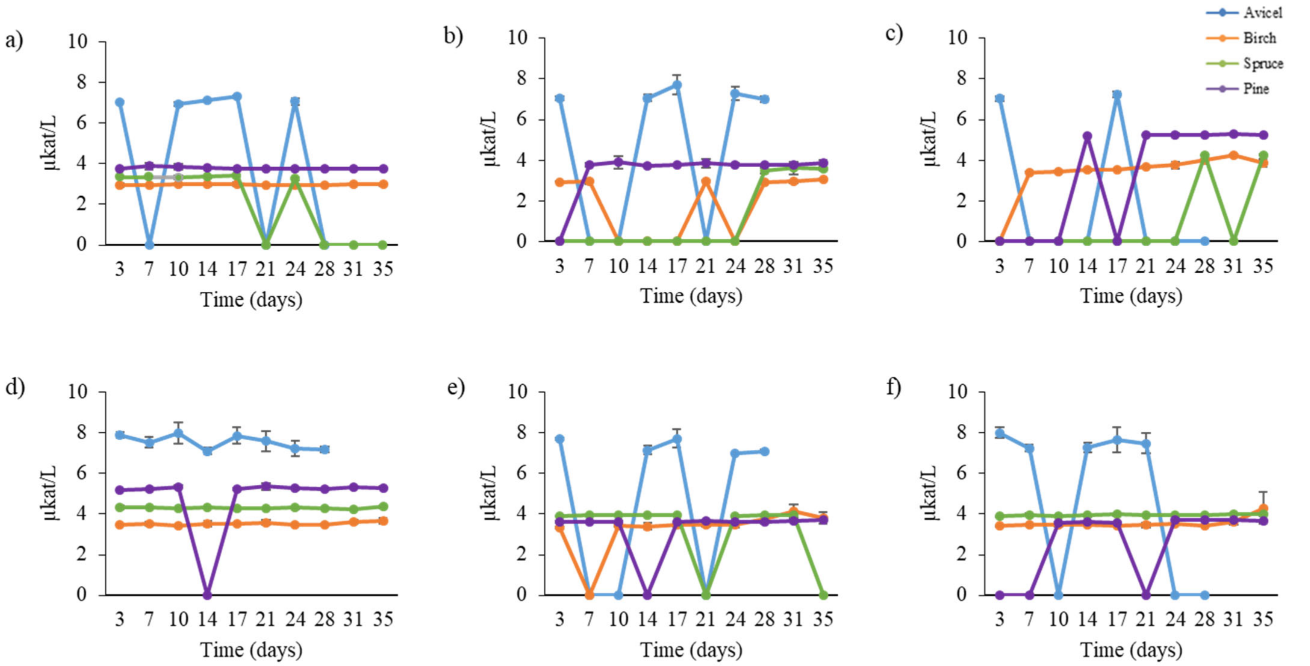

Figure S6. Endomannanase (MAN) activities on woody substrates and Avicel in single cultivations, a) *A. sinuosa*, b) *G. sepiarium*, and c) *B. adusta*, and in co-cultivations, d) *A. sinuosa* and *G. sepiarium*, e) *A. sinuosa* and *B. adusta*, and f) *G. sepiarium* and *B. adusta*. Error bars refer to standard deviation (SD) (n = 3).

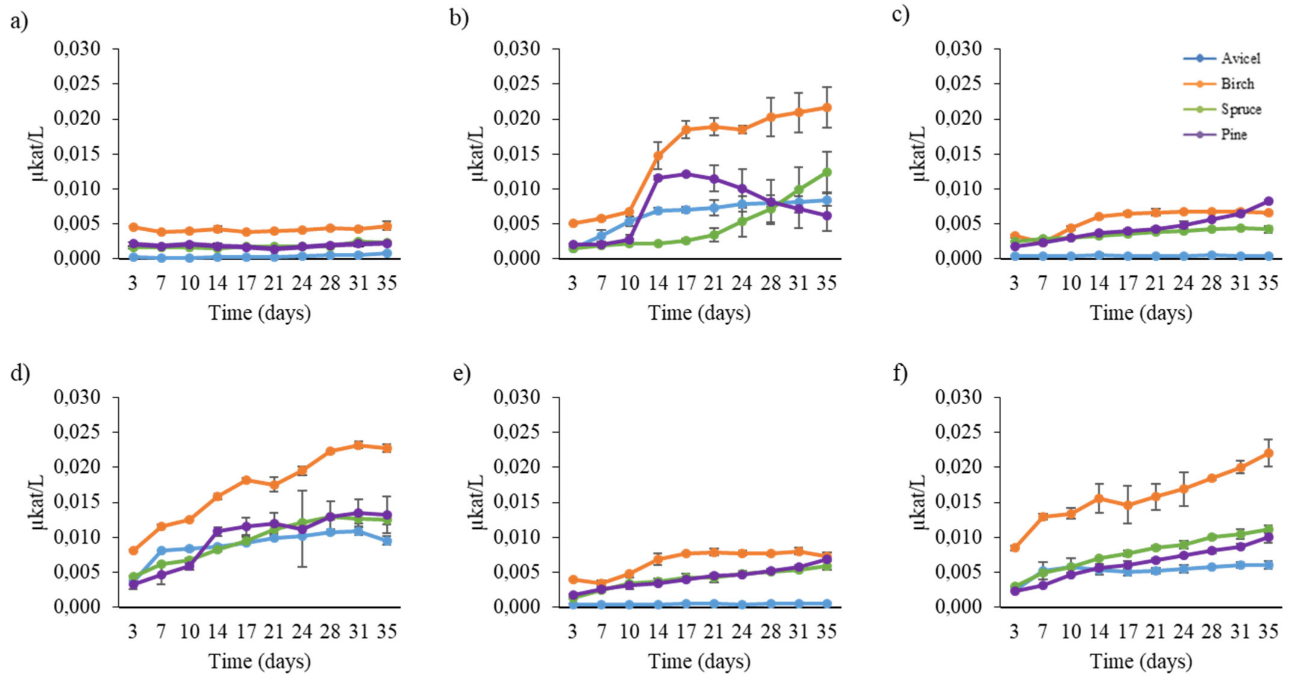

Figure S7. β-mannosidase (MND) activities on Avicel and woody substrates in single cultivations, a) *A. sinuosa*, b) *G. sepiarium*, and c) *B. adusta*, and in co-cultivations, d) *A. sinuosa* and *G. sepiarium*, e) *A. sinuosa* and *B. adusta*, and f) *G. sepiarium* and *B. adusta*. Error bars refer to standard deviation (SD) (n = 3).

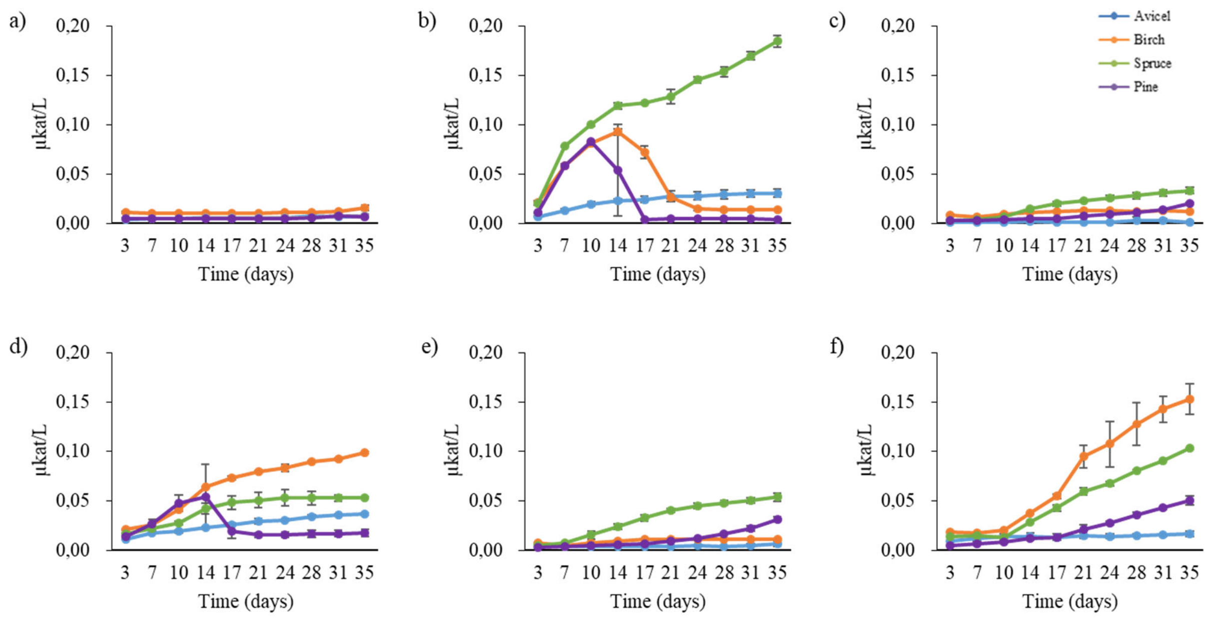

Figure S8. α-galactosidase (AGL) activities on woody substrates and Avicel in single cultivations, a) *A. sinuosa*, b) *G. sepiarium*, and c) *B. adusta*, and in co-cultivations, d) *A. sinuosa* and *G. sepiarium*, e) *A. sinuosa* and *B. adusta*, and f) *G. sepiarium* and *B. adusta*. Error bars refer to standard deviation (SD) (n = 3).

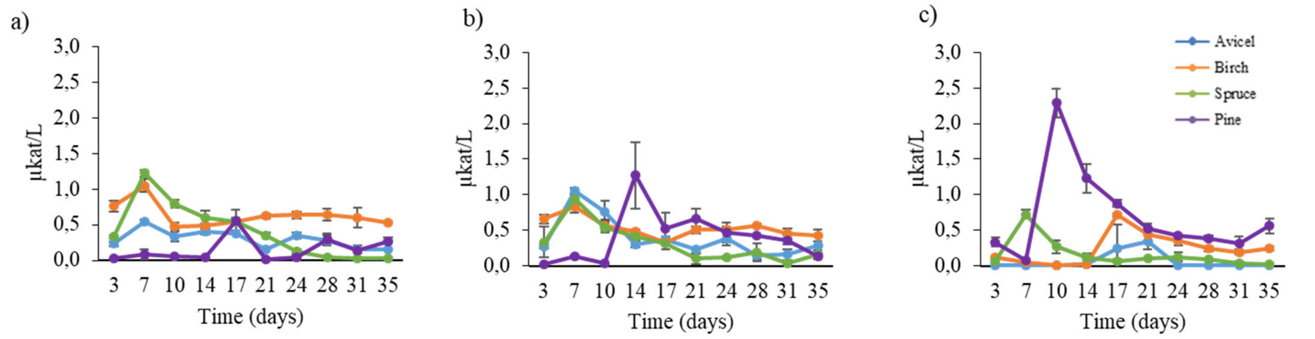

Figure S9. Manganese peroxidase (MnP) activities on woody substrates and Avicel in single cultivations of a) *B. adusta*, and in co-cultivations, b) *A. sinuosa* and *B. adusta*, and c) *G. sepiarium* and *B. adusta*. Error bars refer to standard deviation (SD) ($n = 3$).
